# Febrile neutropenia following with single-low-dose methotrexate for the treatment of ectopic pregnancy: a case report

**DOI:** 10.11604/pamj.2021.38.17.27507

**Published:** 2021-01-07

**Authors:** Burak Bayraktar

**Affiliations:** 1 Department of Obstetrics and Gynecology, University of Health Sciences Tepecik Training and Research Hospital, Izmir, Turkey

**Keywords:** Ectopic pregnancy, methotrexate, medical therapy, pharmacogenetics, case report

## Abstract

Methotrexate (MTX) is an effective, economical and safe drug used in the treatment of ectopic pregnancy. Complications are very rare. Herein, we reported a case of febrile neutropenia following single low-dose methotrexate for the treatment of ectopic pregnancy. Febrile neutropenia developed on day 4 of single-dose methotrexate administered intramuscularly. Although methotrexate single-dose regimen is quite effective and safe in ectopic pregnancy, febrile neutropenia can occur very rarely.

## Introduction

Ectopic pregnancy (EP) is defined as the placement of the fertilized ovum in a field outside the endometrial cavity. It is often seen in the tubal area, as well as in the hysterotomy scar, abdominal, ovarian, cervical, stump line, and rudimentary horn [1]. It accounts for 2% of all recognized pregnancies, and its frequency has been increasing worldwide in recent years [1]. At present, EP is a life-threatening condition and is still one of the most important causes of maternal loss in the first trimester [2].

EP can be treated medically or surgically. The choice of treatment varies depending on the clinical situation, EP localization, and available treatment options. Medical treatment has many advantages over surgical treatment in non-ruptured EP. These include less tubal damage, lower cost, and elevation in subsequent fertility potential. Methotrexate (MTX) is the most preferred agent in medical treatment for EP since the 1980s [1]. MTX is a folic antagonist that prevents deoxyribonucleic acid synthesis by inhibiting dihydrofolate reductase. It affects actively growing cells such as trophoblastic tissues, malignant cells, bone marrow, intestinal mucosa, and respiratory epithelium. The most important factor in the selection of treatment is a low side-effect and high-effectiveness profile. The most common side effects are stomatitis and conjunctivitis. Rare side effects include gastritis, enteritis, dermatitis, pneumonitis, alopecia, elevated liver enzymes, and bone marrow suppression.

For febrile neutropenia, fever by single oral measurement without any environmental factors is defined as temperature > 38.3°C or >38.0°sC for >1 h [3]. Neutropenia is defined as neutrophil level < 500/mm^3^ or neutrophil level between 500 and 1000/mm^3^ and is expected to fall <500/mm^3^ within 48 h [3]. Febrile neutropenia is extremely rare when using a single dose of MTX for EP. Herein, we present the case of febrile neutropenia due to the use of single-dose MTX in the treatment of EP.

## Patient and observation

A 29-year-old pregnant woman (gravida 2, para 1), without other disease and allergy history, presented with complaints of vaginal bleeding and menstrual delay. Based on her last menstrual period, she was 7 weeks pregnant. On gynecological examination, minimal vaginal bleeding was observed, and no additional pathology was noted. There was no defense or rebound in the abdominal examination. Other physical examination findings were heart rate of 72 beats/min and blood pressure of 110/70 mmHg. On transvaginal ultrasonography, the endometrium thickness was 6mm. The gestational sac or suspected mass was not observed in the cavity. The left adnexal area was normal. An 11mm diameter suspected mass was observed in the right adnexal area. The mass was compatible with gestational sac, but the yolk sac and fetal node could not be distinguished. Free fluid was not observed in the abdomen. The patient's beta human chorionic gonadotropin (β-hCG) value was 3155 mIU/mL. The patient was hospitalized with the diagnosis of EP. Her hemogram results were as follows: hemoglobin of 11 g/dL, hematocrit of 35.7%, platelet count of 192.000/mm^3^, leukocyte count of 7200/mm^3^, and neutrophil count of 4900/mm^3^. The patient's coagulation parameters (prothrombin time and international normalized ratio), liver enzymes (alanine aminotransferase, 10 IU/L; aspartate aminotransferase, 16 IU/L; lactate dehydrogenase, 480 IU/L), and creatine (0.8 mg/dL) levels were normal.

**Figure 1:**
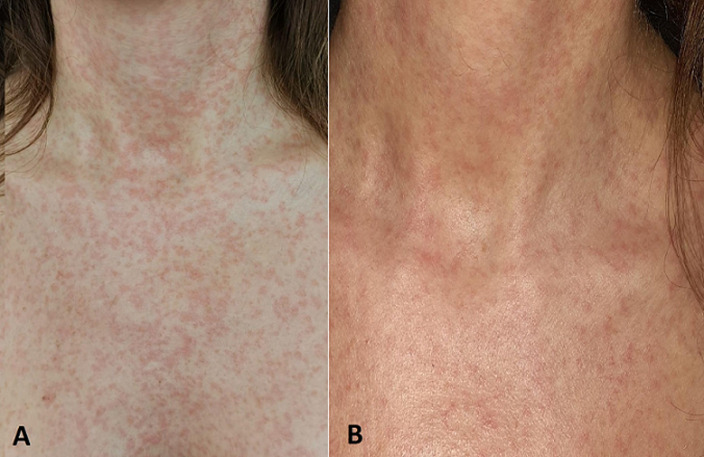
A)generalized mild erythema; B) close-up view of erythema

The patient was treated with a single dose of MTX. A total of 80-mg single-dose MTX was administered intramuscularly according to the 50 mg/m^2^ dose calculation. On day 4 of treatment, generalized mild erythema and mucositis were observed (Figure 1). The patient's temperature was 39°C. On laboratory evaluation, the hemoglobin level was 10.5 g/dL, the hematocrit was 32.3%, and the platelet count was 174000/mm^3^, while the leukocyte count was 4100/mm^3^. The level of β-hCG decreased to 2420 mIU/mL. The neutrophil count of the patient was 700/mm^3^. Thereupon, neutropenic fever was diagnosed. Blood culture was taken from the patient, and empirical intravenous (IV) meropenem 3 x 500 mg and teicoplanin 1 x 400 mg were started. As a granulocyte colony-stimulating factor (CSF), filgrastim 30 million units/day was given subcutaneously, and calcium folinate 4 x 15 mg/day as an MTX antidote was started intravenously. On day 3 of treatment (day 7 of MTX treatment), the leukocyte count was 5700/mm^3^ and the neutrophil count was 2500/mm^3^, which began to rise. The patient did not need transfusion of blood products during follow-up. After 7 days of treatment (day 14 of MTX treatment), the patient's erythema and neutropenia improved and was monitored from the outpatient clinic (Table 1).

**Table 1:** demographic and medical characteristics

Day of treatment	Hb (g/dl)	Hct (%)	Platelet (/mm^3^)	WBC (/mm^3^)	Neu (/mm^3^)	β-hCG (mIU/mL)
**1^st^**	11	35.7	192000	7200	4900	3155
**4^th^**	10.5	32.3	174000	4100	700	2420
**7^th^**	10.6	32.8	180000	5700	2500	1710

Abbreviations: Hb=hemoglobin, Hct=hematocrit, WBC= white blood count, Neu= neutrophil, β-hCG = β-human chorionic gonadotropin

## Discussion

A single-dose MTX treatment is selected at the beginning of tubal EP because single-dose treatment is cheaper, requires less follow-up, does not require folic acid administration, and has fewer side effects with multiple-dose protocols. The rate of resolution reported in the literature is approximately 90% for single dose and multiple-dose regimen, but there is no meaningful difference [4]. In this protocol, MTX is administered at a dose of 50 mg/m2. In a single-dose regimen, follow-up is performed on day 4 and day 7 based on the β-hCG value. A 15% decrease in serum β-hCG concentration between day 4 and day 7 is an important indicator of treatment success [4].

MTX is a folic acid analog and binds to dihydrofolate reductase, reducing thymidylate level, purine synthesis, and cell proliferation. It also inhibits other folate-dependent enzymes such as 5-aminoimidazole-4-carboxamide ribonucleotide transformylase (AICAR) [5]. MTX can be administered orally, subcutaneously, intramuscularly, or intravenously [6]. Contraindications to MTX treatment are a history of severe hypersensitivity to MTX, alcoholism, alcoholic liver disease or other chronic liver disease, immunodeficiency syndromes (overt or laboratory evidence), and preexisting blood dyscrasias (e.g., bone marrow hypoplasia, leukopenia, thrombocytopenia, and significant anemia) [7]. Our patient did not have any of these contraindications.

MTX can cause rare toxicity if used at a low dose. The toxic effect can be divided into two groups: major and minor. Minor toxic effects of MTX are seen in 20%-30% and include headache, stomatitis, malaise, nausea, vomiting, diarrhea, and mild alopecia [8]. The major toxic effects of methotrexate are much less common in minor toxic effects but may be life-threatening. The major toxic effects of methotrexate can be listed as hepatic, renal, pulmonary and bone marrow disorders [8]. Myelosuppression and febrile neutropenia are very rare adverse effects of low-dose MTX. It can be overlooked because of its rarity. Untreated cases may lead to death. Some publications have indicated that methylene tetrahydrofolate reductase (MTHFR) polymorphism may be caused by low-dose MTX toxicity that occurs without a significant risk factor, such as impaired renal function [9, 10]. However, there is no consensus on the type of polymorphism and its evaluation before the first dose of MTX [11], so we did not perform polymorphism analysis in our patient.

Febrile neutropenia should be suspected if fever, generalized erythema, and mucositis are observed during single-dose MTX treatment. Hemogram should be taken quickly. Serial neutrophil follow-up should be carried out, blood culture should be taken, and empirical IV antibiotics should be started. Antibiotics to be given must have an antipseudomonal effect [12]. Calcium folinate is an MTX antidote and is easily available, so it should be administered. The addition of CSFs to treatment in febrile neutropenia reduces the duration of neutropenia, but does not have a positive effect on morbidity, fever duration, IV administration of antibiotics, and treatment cost [13]. For this reason, a granulocyte CSF can be used in appropriate cases, but it does not have known significant effect on morbidity.

## Conclusion

Single-dose MTX therapy is used effectively in the treatment of EP. Although rare, it may be associated with myelosuppression and febrile neutropenia. Therefore, clinicians using MTX for this indication should be aware of this potential complication and act accordingly.
